# Taka Oguisso’s investiture rites at the Brazilian Academy of Nursing History: 1st Academician

**DOI:** 10.1590/0034-7167-2025-0106

**Published:** 2025-12-08

**Authors:** Margarete Maria Rodrigues, Fábio Soares de Melo, Alessandra Santos de Paula, Genival Fernandes de Freitas

**Affiliations:** I Universidade de São Paulo. São Paulo, São Paulo, Brazil; II Centro Universitário de Viçosa. Viçosa, Minas Gerais, Brazil

**Keywords:** Ceremonial Behavior, Academies, History of Nursing, Professional Identification, Nursing., Conducta Ceremonial, Academias, Historia de la Enfermería, Identificación Profesional, Enfermería.

## Abstract

**Objectives:**

to describe and analyze the social relevance of Professor Taka Oguisso’s investiture as the first member of the Brazilian Academy of Nursing History.

**Methods:**

this is a qualitative, biographical, and historical study, using the interview method with the first academician. The material was analyzed from the perspective of content analysis, as outlined by Bardin, and the categories identified were discussed within the theoretical framework of philosopher Claude Dubar.

**Results:**

three categories were identified, namely the social relevance of investiture as an academic member, the creation of a rite of investiture, and signs and symbols in the process of building a professional nursing identity.

**Final Considerations:**

the investiture rite and the symbols used contribute to the process of constructing the academic’s professional identity, promoting recognition and belonging in the organization’s social groups, as well as protecting its members from the risk of losing their identity.

## RESUMEN

**Objetivos:**

describir y analizar la relevancia social de la investidura en el rito de posesión de la profesora Taka Oguisso como primer miembro de la Academia Brasileña de Historia de la Enfermería.

**Métodos:**

estudio de enfoque cualitativo, biográfico e histórico, con el uso del método de entrevista realizada a la primera académica. El material se analizó desde la perspectiva del análisis de contenido según Bardin, y las categorías encontradas se discutieron basándose en el marco teórico del filósofo Claude Dubar.

**Resultados:**

se enumeraron tres categorías, a saber, la relevancia social de la investidura como miembro académico, la creación de un rito de posesión y los signos y símbolos en el proceso de construcción de la identidad profesional de la enfermería.

**Consideraciones Finales:**

El rito de posesión y los símbolos utilizados contribuyen al proceso de construcción de la identidad profesional de la académica, promueven el reconocimiento y la pertenencia a los grupos sociales de la entidad, además de proteger a sus integrantes del riesgo de pérdida de identidad.

## INTRODUCTION

The construction of professional identities is a historical, continuous, and unfinished process, the result of various social interactions^(1-4)^. Regarding nursing, this process includes deconstructions and reconstructions that have taken place in a relationship with society permeated by concepts, prejudices, and stereotypes that still influence the understanding of its meaning as a profession and the legitimization of the practice and the potential inherent in professional practice^(5-7)^.

Few studies have focused on understanding how this construction of a professional identity takes place in nursing, so deepening knowledge about this process contributes to understanding it as a profession^(4,8)^. In this sense, this study aims to contribute to broadening discussions on the subject of the construction of professional identities in nursing, and the history of nursing is the best means of enabling the historical recovery of past events that make it possible to understand the processes of constructing these professional identities.

The historical moment recalled in this study refers to the investiture rite of the first academician of the Brazilian Academy of Nursing History (ABRADHENF). It was founded on August 13, 2010, in the auditorium of the Nursing Professional Improvement Center of the Regional Nursing Council - São Paulo section (COREN-SP), with the aim of promoting advances in the study of the history of nursing in Brazil^(9,10)^. This was a milestone for Brazilian nursing and especially for the field of history, under the leadership of Prof. Dr. Taka Oguisso. Taka Oguisso, who brought together the efforts of national and international researchers and professors - EE-USP (Nursing School of the University of São Paulo), Alfredo Pinto Nursing School/UNIRIO, and Alicante (Spain) - whose first discussions on the creation of an Academy took place at the I Ibero-American Symposium on the History of Nursing (I SIAHE), in São Paulo, in 2007, organized by EE-USP^(11)^.

Her nomination to take up the Academy’s first chair was unanimously approved at an Assembly held on September 28, 2012^(12)^. The investiture ceremony took place on December 6, 2013, at the Ribeirão Preto College of Nursing at the University of São Paulo^(13)^, surrounded by ritualistic elements appropriate to the occasion.

A rite or ritual is understood as a series of formal acts with a symbolic dimension, their own space/time framework, languages, signs, and objects, whose interpretation constitutes a common good for the group. It can also be understood as an institutional rite when it enshrines an established order by leading those transformed in this act to behave as the representation of reality^(14)^.

Therefore, the study assumes that the rituals and symbols that comprise them are capable of influencing the process of constructing professional nursing identities and contributing positively to their internalization and incorporation into nursing care practices.

This study seeks to promote further reflection on the subject, since professional identity is a process that is still under construction and unfinished, and therefore there is still much to discuss. The relationship between the formation of professional identity and its retention has not yet been fully evaluated by the nursing profession^(15)^,and the lack of knowledge about the trajectory of the pioneers of CARING makes it impossible to understand the present in its personal and professional alignments and realignments^(9)^.

## OBJECTIVES

To describe and analyze the social relevance of the investiture ceremony for Professor Taka Oguisso as the first member of the Brazilian Academy of Nursing History.

## METHODS

### Ethical aspects

The ethical precepts of research involving human beings were observed in accordance with Resolution 466/2012 of the Ethics and Research Commission/Ministry of Health (CONEP/MS)^(16)^. The research was approved by the Research Ethics Committee of the School of Nursing of the University of São Paulo.

Free and informed consent was obtained from all individuals involved in the study, in writing.

The interviewee authorized the use of her image and the disclosure of the interview in this study, as she is a well-known figure in this Academy and in Brazilian and global nursing..

### Theoretical and methodological framework

The theoretical framework used in this study was that of Claude Dubar, a French philosopher whose work focused on the construction of professional identities, anchored in the concept that identity is not given as a whole at birth.Instead, its construction takes place gradually and throughout life, in a process that is never finished, and is permeated by social interactions^(1)^.

### Study type

This is a qualitative study with a biographical approach, as it enabled an understanding of the interviewee’s experiences regarding the subject studied. *Biography, as a narrative that tells someone’s life story, is a very widespread textual genre* ^(17)^, and bringing to light the professional trajectory of a personality has the potential to reinforce their relevance to the community and contribute to the construction of professional identity, in the case of nursing.

Qualitative studies are important for healthcare professionals because they allow them to understand human experiences and deepen their knowledge about their nature, especially for nursing, considering that the practice of these professionals is based on interpersonal relationships, communication, and care^(18,19)^.In order to qualify scientific production on the subject, the guidelines of the Consolidated Criteria for Reporting Qualitative Research (COREQ) were adopted^(20)^.

### Methodological procedures

Oral history was the most appropriate option for the development of this study, and it was chosen because it was the only method capable of generating data that would enable a better understanding of the phenomenon^(21)^. In addition, it enabled a better understanding of the interview regarding the social relevance that these initiation rites represent for her and for the community in which she lives^(22,23)^.

### Study setting

The study was conducted at the Brazilian Academy of Nursing History, an organization whose objectives include promoting discussions about teaching, research, and outreach activities in the field of nursing history, thereby contributing to the construction of a collective nursing identity.

Another reason for choosing this location is that it is an academy, and as such, it maintains an attachment to ritualistic traditions in the events it organizes.

The inclusion criterion was to invite the first academician, Professor Taka Oguisso, to participate in the study, because she was a pioneer in mobilizing efforts to found ABRADHENF and in being inducted as the first academician, and because she is a national and international reference in ethics, legislation, and the history of nursing. Thus, she is the “key individual” who could offer the best contributions to this study^(22)^.

### Data source

Documentary and image sources available in the collections of the Cultural Historical Center of Ibero-American Nursing at EE-USP and ABRADHENF were used to support the interview, including the proceedings of the founding, appointment, and investiture assemblies, as well as the image collection of the investiture rite that took place in 2013. All of these documents were analyzed in order to create a body of information to support the interview.

### Data collection and organization

The interview was based on a guiding question: “Could you tell us about your experience of being appointed to the first chair of ABRADHENF?”. Based on this guiding question, topics involving the creation of the investiture rite, the interviewee’s academic and professional career, the symbolic elements involved in the investiture rite, her perception of the event, and its impact on the community were addressed.

The material was collected in March 2017 through an audio-only interview on the premises of EE-USP, when Dr. Taka Oguisso was still working at the institution. The content was transcribed verbatim and then transcreated, resulting in a body of documents to be analyzed according to Bardin^(24)^.

### Data analysis

This stage focused on the research question: “How did and does the collaborator, Prof. Taka Oguisso, perceive the relevance of investiture and the rites of investiture/membership in the Brazilian Academy of Nursing History? And how did and does this perception influence the understanding of the processes of belonging to this organization and the identity construction of becoming an academic member?”.

The first phase consisted of a preliminary review of the accounts, with the intention of becoming familiar with the content of the interview and extracting first impressions of the content. The next phase consisted of exhaustive readings in order to find common characteristics through the coding process, with the hope of finding elements pertinent to the description of the ritualistic processes practiced by the academy, elements about the interviewee’s perception of the ritualistic process, as well as the importance of these rites for the identity construction process of the academy’s members. At this stage, categories were also constructed according to the similarities between the units of records.

In the last phase, the categories obtained were submitted to the interpretative process in the light of the theoretical framework of the construction of professional identity according to Claude Dubar.

## RESULTS

The results of this research pointed to three categories considered relevant and which reflect the importance of rites, symbols, and signs in the construction of professional nursing identity.

### Social relevance of becoming an academic member

When asked how she felt about being appointed to her first chair as an academic member, the interviewee was emphatic, highlighting the responsibility of taking on such a position (interviewee) and the joy of being recognized by her colleagues (interviewee). She also emphasized: [...] *I take this gratification much more as a responsibility* (interviewee).

She also stressed her responsibility to continue being an example to other colleagues and professionals in the field and recalled the figure of the patron of ABRADHENF’s first chair, Prof. Dr. Amália: [...] *she gave me this example, and I want to leave this example for future generations. New nurses, future colleagues, those who are about to enter or have already entered the profession. We have to keep this light on, this ideal in front of us* (interviewee).

Concluding her thoughts, she stated that this is the most important point when accepting and taking on a position like this, which involves *a position of deference from your own colleagues* (interviewee).

The ceremony had repercussions in other organizations representing the professional category, such as the Federal Nursing Council^(25)^, which published the following note on its website: *ABRADHENF appointed the first female nursing academician, an unprecedented fact in the history of Brazilian nursing.*

### Creating an investiture rite

The interview made it possible to recall, through her memories, the process of creating the ceremonial by which she was nominated into office:

*The first thing Luciana asked me to do was to have a cloak made, the way the cloak used to be. I wore this cloak as a student; I have photos of myself wearing it. In my day, here at school, I also had a cloak, so much so that I put the cloak on the mannequin in the corridor.* (interviewee)

The characterization of the other members of the ABRADHENF board of directors did not go unnoticed by the organizers of the ceremony and all of them, as the interviewee recalled, were dressed *as if they were graduating* (interviewee).

The interviewee continued to narrate the development of the rite: *I entered with Sister Tereza because she is a historical figure* [...] *and then Luciana arranged the stage with a chair* [...] *for me to sit on this day* (interviewee).

She continued: *Well, then there was this ceremony in which the entire board of directors in the auditorium granted me the title of academician of the Brazilian Academy of Nursing History* (interviewee).

The auditorium to which the interviewee referred is located at the Ribeirão Preto College of Nursing at the University of São Paulo, the venue chosen for the investiture ceremony. The choice of city was no coincidence: it is the hometown of the patron of the academy’s first chair, Prof. Amália Correa de Carvalho.

He further concluded:

*That was the ceremony where I gave a speech, and six weeks earlier, Fernando Henrique Cardoso had taken up his seat at the Brazilian Academy of Letters, and I used his words because I thought it was appropriate, as he spoke of a rite. He was talking exactly about a rite and we were following a rite there, we were creating a rite for this ceremony to appoint an academic member of the Academy.* (interviewee)

### Signs and symbols in the process of building a professional nursing identity

When asked about the importance of the clothing worn at the ceremony, the interviewee established a relationship of equality between the cloak she wore and the toga worn by the Supreme Court authorities:

*I think, for example, the Supreme Court. You see that when they appear on television, when they are in session, in judgment, they all wear the toga. What is the symbolism of the toga? What does the toga mean? It’s a symbol of authority* [...]*. So this is a rite, isn’t it? It’s the same with the Academy, so the cloak represents the judge’s toga.* (interviewee)

Continuing her reflection on the cloak used, she added:

*The cloak represents the Supreme Court judge’s toga. So it represents or is intended to represent this symbol of the Academy of Nursing, which no other entity, neither COFEN, nor ABEN, nor union, nor Federation gives it. They don’t use it, do they? But the Academy does. It is a rite.* (interviewee)

**Figure 1:**
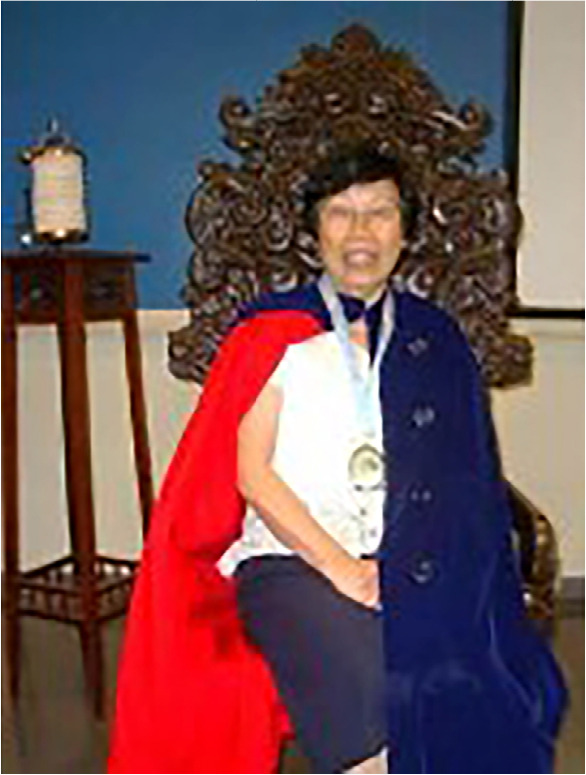
Investiture ceremony at the Brazilian Academy of Nursing History

Regarding the colors of the cloak and its symbolism, upon examining the photographic image (Figure 1), the interviewee revealed that it was a cloak that had been used since the time of Florence Nightingale and remained in use until the mid-1960s. The colors blue on the outside and red on the inside were used by professors at solemn moments, such as graduations or when they were appointed academic members of ABRADHENF.

Figure 1 also reveals that the interviewee wore a medal on her chest attached to a blue ribbon. When asked about the symbolism of this object, she explained that it was a medal with the image of the lamp used by Florence Nightingale.

During the interview, other symbolic elements representing professional nursing were also mentioned, such as the use of caps:

*When I was a student, we had a cap ceremony* [...]*. In the second semester of the first year, we started our internship at the hospital. Before starting the internship, the students received their caps, which carried a whole symbolism behind them. It was a meaningful ceremony because the students received the caps from the principal, who placed them on their heads.* (interviewee)

She continued her recollection of the event, and at this point, the interviewee used another equally significant analogy in relation to the cap: *The school principal said, “You will receive the cap in the same way that a queen receives her crown. When she is crowned queen and receives the crown on her head, then you will receive the cap, which has the same meaning”* (interviewee).

At another point in the interview, the importance of the passing of the lamp ritual in the graduation ceremony was highlighted, and this same symbol was also present in the academic inauguration ceremony:

*And at the end of the course, there was a ceremony, where we would graduate, and I received the lamp in my first year, at the end of the first year, from the student who had gotten the best grade, which was passed on to the student who had gotten the best grade in the first year, based on their average. So I received the lamp from the hands of the colleague who was graduating that year, and then, at the end of the last year, I passed the lamp on to a first-year student.* [...] *It was a ceremony like that, how can I say, it’s a historical thing, actually, I mean, but it had that meaning for us. I mean, so the nurse who was graduating, who was already a nurse, passed it on to the first-year student who was starting her career.* (interviewee)

She concluded:

[...] *It’s not just the cloak, I mean, it’s all the symbolism because we want to emphasize that nursing is more than just working in a hospital. There are things that I think represent, I wouldn’t say power, but they represent our efforts as nursing warriors. To take the light of nursing further, higher, through these symbols.* (interviewee)

## DISCUSSION

The construction of professional identity is not an isolated process, but rather the result of constant socialization^(1)^. It allows individuals to interact with social groups, assimilating the values and culture of these groups and fostering a sense of belonging in the individual. After all, recognizing oneself as a member of a social group is essential in the process of building professional identities, and this is very clear in the interviewee’s statement: *recognition by one’s colleagues* (interviewee).

Recognition by the group is an essential element in social interactions and, according to Dubar^(1)^, the individual, in turn, needs to recognize themselves within that group in order to awaken an interest in being part of it, initiating the process of trying to belong, a movement that will influence the process of constructing their professional identity.

It is in social relationships that individuals have their professional identity influenced and, in turn, affect the professional identity construction of other individuals: [...] *She gave me this example, and I want to pass it on to future generations. New nurses, future colleagues* [...] (interviewee). According to Dubar^(1)^, identity constructions are dynamic and unfinished processes that operate more or less under a climate of instability. This instability is fundamental for these constructions to be questioned, reviewed, and reconstructed by individuals at all times.

In the case of the interviewee, it is noteworthy that the name of Prof. Amália Correa de Carvalho, the personality chosen to name the first chair occupied by Prof. Taka Oguisso, was a nurse who influenced the academician’s professional career, and her choice revives and keeps alive the memory and achievements of this figure as an inspiration for good practices in nursing. Today, Prof. Taka Oguisso’s biography serves as an inspiration to her peers, earning her recognition and a prominent position in the social group within the Academy, making her a pioneer as the first academic member of ABRADHENF.

Her unanimous approval, as recorded in the proceedings of the Assembly^(12)^, reveals that the social group endorses and recognizes the relevance of this social actor to the community, and the following excerpt demonstrates that the honoree is aware of her significance to the group: *So I think that’s the most important poin*t [...] *you accept and take on a position like this, which is a position of deference from your colleagues themselves* [...] (interviewee).

Therefore, professional identity constructions are necessarily mediated by socialization, and the movements undertaken by individuals in their search for recognition and belonging to social groups are decisive for the processes of constructing their identities.

The inauguration ceremony, pioneered by ABRADHENF, has an important meaning for the social group involved, as it was the first of its kind: [...] *We were following a ritual there, creating a ritual for this ceremony to appoint an academic member of the Academy* (interviewee). This collective construction of an inauguration ritual demonstrates the harmony among its members and reinforces the professional identity of each one of them, establishing engagement in what Dubar^(1)^ calls “recognition of social utility”. Everyone present shared the social relevance of the ritual that was being created and would serve as a model for other inaugurations.

Carefully planned and prepared, with a refined selection of elements, the ceremony was permeated with symbols whose meanings renewed the ideal of “being a nurse” for the honoree and other social actors. “Being a nurse” is deeply related to professional identity construction, and Dubar^(1)^ says that institutions must protect their members from the risks of identity loss and that the greater the risk, the more elaborate the rituals should be.

In this context, ritual is understood as a formalized set of symbolic actions, characterized by a spatial-temporal configuration and the use of a series of objects, language systems, behaviors, and signs that need to be decoded in order to be understood as the common good of the group^(26)^.

The revival of the professional identity of nursing began even before the ceremony itself, with the creation of a cloak that the honoree would wear at the investiture ceremony. This was not just any garment, but a costume worn only by nurses until the mid-1960s. In this sense, it revives a symbol of nursing for the new generations of nurses present at the ceremony, as it portrays a moment in the history of nursing when this cloak served as an identifier for other professionals and patients that the wearer was a nurse. The garment gave the wearer an unmistakable professional identity and, at the same time, set them apart^(27)^.

The analogy made by the academician between the cloak she wore at the ceremony and the togas worn by judges reveals the symbolism attributed to clothing and the importance of the person wearing it. Just as the toga worn by a judge qualifies him as the highest authority in matters of law, the garment worn by the honoree qualifies her as an authority in the field of nursing history. Here we note the emergence of new representations attributed to clothing, previously an identifying element of a nursing professional, now also representing established authority, which corroborates Dubar’s^(1)^ idea that representations are not transmitted and constituted once and for all, but are constructed in the form of periodic rearrangements resulting from new assimilations of elements taken from various sectors of the environment.

Other symbols of nursing were revived by the academician, such as the cap and the ritual involving its delivery to the graduating student, as well as the ritual of passing the lamp. All are nationally known in nursing and represent an unmistakable professional identity: nursing. Thus, the recovery of these material and immaterial symbols by the academician - in the latter case, the reference to Prof. Amália and Sister Tereza Notarnicola - corroborates to reinforce the identity of nursing and transform the professional identities of all those present, since, according to Dubar^(1)^,socialization becomes a process of construction, deconstruction, and reconstruction of identities, especially in the professional sphere, where each person must learn to become an actor.

The atmosphere surrounding the academician’s inauguration ceremony had a transformative effect on the honoree’s professional identity, [...] *I take this gratification much more as a responsibility* (interviewee), demonstrating Dubar’s theory that professional identity is an unfinished process in which socialization is a means for such identities to be constantly revisited, deconstructed, and reconstructed. All the elements used in the ceremony have a transformative symbolism for the identities present there. The use of these elements in the ceremony corroborates the assertion that they need to be part of the social group’s universe of meaning in order to be decoded and assimilated as a common good^(26)^.

The first academician was appointed on September 28, 2012, according to the proceedings of the General Assembly^(12)^ of the entity, based on the rules established by the Institutional Statute^(28)^, which establishes four categories of members, including Academician, and by the Internal Regulations for Academic Members^(29)^, defines the criteria for an associate to apply for membership as an Academic Member. These documents stipulate hierarchies among these categories of members, with Academic Member being the one that confers the highest status and is therefore the most important to achieve within the social group. Belonging to this select group awakens in the other members a search for belonging which, according to Dubar^(1)^, are movements in which the individual(s) of a given group seek(s) the necessary means to belong to another group, the reference group, whose primary identification in the group of belonging is that which links the individual to their status.

Delving deeper into these documents, the Institutional Statute^(28)^ provides that full members may be academicians if they are prominent in research and publications in the field of nursing history, according to criteria of quantity and quality of production. It is clear that intellectual production is valued within the Academy and, according to Dubar^(1)^, this means that individuals who wish to move up from the group to which they belong to the reference group within the institution seek qualification as a means of being accepted into that group, whose main identifying feature is the link between the individual and the knowledge acquired.

Another issue concerns institutional documents and is directly related to the professional identity of nursing, specifically the fact that the Academy Statute^(28)^ allows the affiliation of non-nursing professionals or researchers, such as historians, provided that they conduct historical studies (Article 5). This position is corroborated by the Internal Regulations for Academic Members^(29)^, which do not establish the prerogative that only nurses can apply and be nominated for the category of academic member. This means that non-nurse members may apply for the category of full academic members, considering the candidate’s relevant contribution and academic production in the field of nursing history.

The admission of other professions to the Academy has positive implications for the identity building of the Brazilian Academy of Nursing History, considering that the construction of professional identity, according to Dubar^(1)^, occurs through the process of looking at oneself and being looked at by others through social relationships.

For Dubar^(1)^, the reproduction of identities is based on the extreme importance of recognition within social relationships. The entry of a non-nurse professional into the Academy could lead to what Dubar^(1)^ calls a “crisis of social recognition space”, characterized as an institutional environment where social relationships can produce behaviors capable of blocking the construction of social spaces of recognition.

Another relevant aspect refers to institutional rituals, which aim to create an environment in which professional identities can be reinforced and protected, thereby preventing the loss of identity^(1)^. However, for this to happen, all elements, symbols, and signs used in the ritual need to be decoded so that they can be understood as the common good of the group^(26)^. The organization needs to ensure that non-nursing professionals carry out this decoding process properly, producing a universe of meanings that contributes to the construction of their professional identity.

Finally, issues involving identification processes are at stake, given that this element is essential in social relations within the group or between groups within the institution. Identification is what drives social actors’ desire to be part of this or that social group, in a process called “mobility” by Dubar^(1)^. This non-nursing professional needs to identify with the reference group within the Academy, the group of academicians, so that they can imbue themselves with the desire to belong to it through the production of research and publications in the field of nursing history, according to criteria of quantity and quality of production^(28)^.

### Study limitations

This study examines the process of professional identity construction, with a focus on leadership relevant to Brazilian nursing.

Given that professional identity construction is individual, despite being influenced by socialization, it is not possible to establish generalizations for other identity constructions of social actors at the Academy.

Further studies are needed to identify how such identity constructions occur.

### Contributions to the field of nursing

The findings contribute to fostering discussions in nursing history and professional identity, as well as making a relevant contribution to reinforcing the importance of institutional rites for the construction of professional identities in nursing and for strengthening these identities.

## FINAL CONSIDERATIONS

Rites, their signs, symbols, and representations constitute a relevant part of the processes that favor the construction of professional identities in nursing, insofar as they instill in individuals the idea of belonging to the social groups of which they are a part. They also imply recognition of the historical professional and social trajectory of this social actor by the collective.

The inauguration ceremony was important for keeping alive the memory and meaning of “being a nurse” from the perspective of the academician referred to here, renewing her commitment to nursing.

It is essential to remain attentive to conditions that may favor the loss of identity, given that the construction of professional identity is a dynamic, unfinished process in which social relations interfere at every moment.

## Data Availability

The research data are available within the article.
