# Supplementary material for: Taka Oguisso’s investiture rites at the Brazilian Academy of Nursing History: 1st Academician
Source: Rev Bras Enferm. 2025 Dec 8;78(5):e20250106. doi: 10.1590/0034-7167-2025-0106 (PMC12707755; doi:10.1590/0034-7167-2025-0106)
Supplement: Translation

# Translation: Ritos de investidura de Taka Oguisso na Academia Brasileira de História da Enfermagem: 1ª Acadêmica

**DOI:** 10.1590/0034-7167-2025-0106pt

**Authors:** Margarete Maria Rodrigues, Fábio Soares de Melo, Alessandra Santos de Paula, Genival Fernandes de Freitas

**Affiliations:** I Universidade de São Paulo. São Paulo, São Paulo, Brasil; II Centro Universitário de Viçosa. Viçosa, Minas Gerais, Brasil

**Keywords:** Rituais, Academia, História da Enfermagem, Identificação Profissional, Enfermagem.

## Abstract

**Objetivos:**

descrever e analisar a relevância social da investidura no rito de posse da professora Taka Oguisso como primeiro membro da Academia Brasileira de História da Enfermagem.

**Métodos:**

estudo de abordagem qualitativa, biográfica e histórica, com uso do método de entrevista realizada com a primeira acadêmica. O material foi analisado sob a ótica da análise de conteúdo segundo Bardin, e as categorias encontradas foram discutidas com base no referencial teórico do filósofo Claude Dubar.

**Resultados:**

três categorias foram elencadas, a saber, relevância social da investidura como membro acadêmico, criação de um rito de posse e signos e símbolos no processo de construção da identidade profissional da enfermagem.

**Considerações Finais:**

o rito de posse e os símbolos utilizados contribuem no processo de construção da identidade profissional da acadêmica, promove reconhecimento e pertencimento nos grupos sociais da entidade, além de proteger seus integrantes do risco de perda de identidade.

## INTRODUÇÃO

A construção das identidades profissionais é um processo histórico, contínuo e inacabado, fruto de várias interações sociais^(1-4)^. Em relação à enfermagem, acrescenta-se a esse processo desconstruções e reconstruções ocorridas numa relação com a sociedade permeadas por conceitos, preconceitos e estereótipos que influenciam até hoje na compreensão do seu significado como profissão e na legitimação da prática e das potencialidades inerentes ao exercício profissional^(5-7)^.

Poucos estudos têm se ocupado em compreender como se dá essa construção identitária profissional na enfermagem, portanto, aprofundar os conhecimentos sobre esse processo contribui para a compreensão da mesma enquanto profissão^(4,8)^. Nesse sentido, o presente estudo pretende contribuir para a ampliação das discussões acerca da temática da construção das identidades profissionais da enfermagem, sendo que a História da Enfermagem se constitui no melhor meio para viabilizar o resgate histórico de momentos do passado que possibilitem a compreensão dos processos de construção dessas identidades profissionais.

O momento histórico resgatado neste estudo refere-se ao rito de posse da primeira acadêmica da Academia Brasileira de História da Enfermagem (ABRADHENF). Entidade fundada em 13 de agosto de 2010, no auditório do Centro de Aprimoramento Profi-ssional da Enfermagem do Conselho Regional de Enfermagem - seção São Paulo (COREN-SP), com o objetivo de promover avanços nos estudos da história da enfermagem no Brasil^(9,10)^. Um marco para a enfermagem brasileira e principalmente para o campo da História, sob a liderança da profª Dra.Taka Oguisso, que aglutinou esforços de pesquisadores e professores nacionais e internacionais - EE-USP (Escola de Enfermagem da Universidade de São Paulo), Escola de Enfermagem Alfredo Pinto/UNIRIO e Alicante(Espanha) - cujas primeiras discussões sobre a criação de uma Academia ocorreram no I Simpósio Ibero-Americano de História da Enfermagem (I SIAHE), em São Paulo, em 2007, organizado pela EE-USP^(11)^.

Sua indicação para assumir a primeira cadeira da Academia foi aprovada com unanimidade, em Assembleia realizada em 28 de setembro de 2012^(12)^. A cerimônia de empossamento ocorreu no dia 06 de dezembro de 2013, na Escola de Enfermagem de Ribeirão Preto da Universidade de São Paulo^(13)^, cercada por elementos ritualísticos próprios para a ocasião.

Entende-se por rito ou ritual uma série de atos formais com dimensão simbólica, recorte espaço/tempo próprios, linguagens, signos e objetos próprios cuja interpretação constitui um bem comum ao grupo mas também pode ser entendido como um rito institucional, quando consagra uma ordem estabelecida ao levar aqueles transformados nesse ato a se comportarem como a representação do real se mostra^(14)^.

Assim sendo, o estudo tem como pressuposto que os ritos e os símbolos que os integram são capazes de influenciar no processo de construção das identidades profissionais da enfermagem e contribuir positivamente para sua interiorização e incorporação nas práticas de cuidado de enfermagem.

Busca-se, neste estudo, fomentar ainda mais as reflexões acerca da temática, uma vez que a identidade profissional é um processo ainda em construção e inacabado, portanto, ainda há muito para se discutir. A relação entre a formação da identidade profissional e sua retenção ainda não foi totalmente avaliada pela profissão de enfermagem^(15)^, bem como o desconhecimento da trajetória percorrida pelos pioneiros e pioneiras do CUIDAR torna impossível entender o presente nos seus alinhamentos e realinhamentos pessoais e profissionais^(9)^.

## OBJETIVOS

Descrever e analisar a relevância social da investidura no rito de posse da professora Taka Oguisso como primeiro membro da Academia Brasileira de História da Enfermagem.

## MÉTODOS

### Aspectos éticos

Foram observados os preceitos éticos da pesquisa envolvendo seres humanos de acordo com a Resolução da Comissão de Ética e Pesquisa/Ministério da Saúde (CONEP/MS) 466/2012^(16)^. A pesquisa foi aprovada pelo Comitê de Ética em Pesquisa da Escola de Enfermagem da Universidade de São Paulo.

O Consentimento Livre e Esclarecido foi obtido de todos os indivíduos envolvidos no estudo, por meio escrito.

A entrevistada autorizou o uso de sua imagem bem como a divulgação da entrevista nesta pesquisa, já que se trata de um nome com notória visibilidade nesta Academia, na enfermagem brasileira e mundial.

### Referencial teórico-metodológico

O referencial teórico usado neste estudo foi o de Claude Dubar, filósofo francês que teve como cerne dos seus trabalhos a questão da construção das identidades profissionais, ancorado no conceito de que a identidade não é dada como um todo no nascimento, sua construção acontece gradativamente e ao longo da vida, num processo nunca acabado, e permeado por interações sociais^(1)^.

### Tipo de estudo

Trata-se de um estudo qualitativo com abordagem biográfica, pois possibilitou a compreensão das vivências da entrevistada acerca do objeto estudado. *A biografia, enquanto narrativa que conta a história de vida de alguém, é um gênero textual muito difundido^(^* ^17)^, e trazer à luz esta trajetória profissional de uma personalidade tem potencial para reforçar sua relevância para a coletividade e contribuir para a construção identitária profissional, no caso da enfermagem.

A importância dos estudos qualitativos é relevante para os profissionais de saúde por possibilitarem a compreensão das experiências humanas e o aprofundamento no conhecimento sobre sua natureza, principalmente, para a enfermagem, considerando-se o fato de que a prática desses profissionais está pautada nas relações interpessoais, na comunicação e no cuidado^(18,19)^.Com o intuito de qualificar a produção científica acerca da temática, foram adotadas as diretrizes do *Consolidated Criteria for Reporting Qualitative Research* (COREQ)^(20)^.

### Procedimentos metodológicos

A história oral foi a opção mais adequada para o desenvolvimento deste estudo, e sua escolha deu-se por ser a única capaz de gerar dados que possibilitam compreender melhor o fenômeno^(21)^. Além disso, possibilitou a captação de uma melhor compreensão da entrevista acerca da relevância social que esses ritos de investidura representam para ela e para a coletividade na qual está inserida^(22,23)^.

### Cenário do estudo

O local para a realização do estudo foi a Academia Brasileira de História da Enfermagem, entidade que tem como um dos seus escopos o de fomentar discussões acerca do ensino, da pesquisa e das atividades de extensão no campo da História da Enfermagem, contribuindo para alavancar a construção da identidade do coletivo da enfermagem.

Soma-se aos motivos de sua escolha o fato de ser uma Academia e, como tal, manter apego às tradições ritualísticas nos eventos por ela praticados.

Adotou-se, como critério de inclusão, convidar, para participar da pesquisa, a primeira acadêmica, Porfª Drª Taka Oguisso, pelo pioneirismo em mobilizar esforços para a fundação da ABRADHENF, pioneirismo em ser empossada como primeira acadêmica e por ser uma referência nacional e internacional em Ética, Legislação e História da Enfermagem. Assim, é o “indivíduo chave” que se constitui naquele que poderia oferecer as melhores contribuições para este estudo^(22)^.

### Fonte de dados

Foram utilizados, como material de suporte à entrevista, fontes documentais e imagéticas disponíveis nos acervos do Centro Histórico Cultural da Enfermagem Ibero-Americana da EE-USP e da ABRADHENF, tais como, as Atas das Assembleias de fundação, de indicação da acadêmica e de empossamento da mesma, bem como acervo imagético do rito de posse ocorrido em 2013. Tais documentos foram analisados com o propósito de constituir um corpo de informações que subsidiou a entrevista.

### Coleta e organização dos dados

A realização da entrevista pautou-se em uma questão norteadora, a saber: “A senhora poderia relatar sua experiência sobre ter sido indicada para assumir a primeira cadeira da ABRADHENF?”. E, a partir dessa questão norteadora, foram abordados temas envolvendo a criação do rito de posse, a trajetória acadêmica e profissional da entrevistada, os elementos simbólicos envolvidos no rito de posse, sua percepção sobre o evento e os reflexos do evento para a coletividade.

A coleta do material ocorreu em março de 2017, em entrevista gravada somente em áudio, nas dependências da Escola de Enfermagem da EE-USP, período no qual a Porfª Drª Taka Oguisso ainda frequentava a instituição. O conteúdo foi transcrito literalmente e depois transcriado, resultando no corpo documental a ser analisado segundo Bardin^(24)^.

### Análise dos dados

Essa etapa manteve como foco a pergunta de pesquisa: “Como a colaboradora, Profª Drª Taka Oguisso, percebeu e percebe a relevância da investidura e dos ritos de posse/pertencimento na Academia Brasileira de História da Enfermagem? E como essa percepção influenciou e influencia na compreensão dos processos de pertencimento a essa entidade e na construção identitária do tornar-se membro acadêmico?”.

A primeira fase constituiu-se na realização de uma leitura flutuante dos relatos com a intenção de familiarizar-se com o conteúdo da entrevista e extrair as primeiras impressões sobre o conteúdo. A fase seguinte consistiu em exaustivas leituras, a fim de buscar características comuns por meio do processo de codificação, sendo esperado encontrar elementos pertinentes à descrição dos processos ritualísticos praticados pela academia, elementos sobre a percepção da entrevistada a respeito do processo ritualístico, bem como a importância desses ritos para o processo de construção identitária dos membros da academia. Nessa etapa, também foram construídas as categorias segundo as semelhanças entre as unidades de registros.

Na última fase, as categorias obtidas foram submetidas ao processo interpretativo à luz do referencial teórico da construção da identidade profissional segundo Claude Dubar.

## RESULTADOS

Os resultados desta pesquisa apontaram três categorias consideradas relevantes e que refletem a importância dos ritos, símbolos e signos para a construção da identidade profissional da enfermagem.

### Relevância social da investidura como membro acadêmico

Perguntada sobre como se sentiu ao ser indicada para assumir a primeira cadeira como membro acadêmico, a entrevistada foi enfática, destacando a *responsabilidade de assumir uma posição como essa* (entrevistada)e a alegria do *reconhecimento dos próprios colegas* (entrevistada). Enfatizou ainda: [...] *eu assumo essa gratificação muito mais como uma responsabilidade* (entrevistada).

Destacou também a responsabilidade de continuar sendo exemplo para os demais colegas e profissionais da área e resgatou a figura da patrona da primeira cadeira da ABRADHENF, a Profa. Dra. Amália: [...] *ela me passou esse exemplo, e eu quero deixar esse exemplo para as futuras gerações. Os novos enfermeiros, os futuros colegas, enfim, que vão ou estão ingressando ou já ingressaram na profissão. A gente tem que manter uma luz acesa, esse ideal na frente* (entrevistada).

Concluindo seu pensamento, referiu *que esse é o ponto mais importante quando você aceita e assume uma posição como essa que é uma posição de deferência dos próprios colegas* (entrevistada).

A cerimônia teve repercussão em outras entidades representativas da categoria profissional, como no caso do Conselho Federal de Enfermagem^(25)^, que publicou, em seu site, a seguinte nota: *foi investida pela ABRADHENF a primeira acadêmica de enfermagem, fato inédito na história da enfermagem brasileira.*

### Criação de um rito de posse

Pela entrevista, foi possível resgatar, por suas lembranças, o processo de criação do cerimonial por meio do qual foi empossada:

*A primeira coisa, a Luciana me pediu foi que mandasse fazer uma capa, como era a capa antigamente. Eu cheguei a usar, como aluna, essa capa, eu tenho fotos em que estou com essa capa. No meu tempo, aqui na escola, também tinha capa, tanto que eu coloquei a capa ali no manequim que fica no corredor.* (entrevistada)

A caracterização dos demais componentes da diretoria da ABRADHENF não passou despercebida aos olhos dos organizadores do cerimonial e todos, conforme retratou a entrevistada, vestiram beca *como se fosse uma formatura* (entrevistada).

A entrevistada continuou a narrar o desenvolvimento do rito: *Eu entrei com a irmã Tereza por ser uma figura histórica* [...] *e aí a Luciana arrumou o palco com uma cadeira [...]para que eu me sentasse neste dia* (entrevistada).

Continuou: *Bom, e aí houve essa cerimônia em que a diretoria inteira composta no auditório me concedeu esse título de acadêmica da Academia Brasileira de História da Enfermagem* (entrevistada).

O auditório a que a entrevistada se referiu é localizado na Escola de Enfermagem de Ribeirão Preto da Universidade de São Paulo, local escolhido para a cerimônia de posse. A escolha da cidade não foi por acaso, trata-se da cidade natal da patrona da primeira cadeira da academia, a profa. Dra. Amália Correa de Carvalho.

Concluiu ainda:

*Então essa foi a cerimônia onde fiz um discurso e, seis semanas antes, o Fernando Henrique Cardoso tinha assumido a cadeira na Academia Brasileira de Letras, eu usei as palavras dele porque achei que caía bem porque ele falava em rito. Ele falava exatamente em rito e nós estávamos seguindo um rito ali, nós estávamos criando um rito para essa cerimônia de designação de um membro acadêmico da Academia.* (entrevistada)

### Signos e símbolos no processo de construção da identidade profissional da enfermagem

Ao ser perguntada sobre a importância da vestimenta usada na cerimônia, a entrevistada estabeleceu uma relação de igualdade entre a capa usada por ela e a toga usada pelas autoridades do Supremo Tribunal Federal:

*Eu acho que, por exemplo, o Supremo Tribunal Federal. Você vê que quando eles aparecem na televisão, quando eles estão em sessão, em julgamento, todos eles vestem a toga. Qual o simbolismo da toga? O que significa a toga? É símbolo de autoridade* [...]*. Então isto é um rito, não é? A mesma coisa com relação à Academia, quer dizer, então a capa representa a toga do juiz.* (entrevistada)

Continuando sua reflexão acerca da capa usada, complementou:

*A capa representa a toga do juiz do Supremo Tribunal. Então a capa representa ou quer representar esse símbolo da Academia de Enfermagem, que nenhuma outra entidade, nem COFEN, nem ABEN, nem sindicato, nem a Federação dá. Não usa isto, não é? Então a Academia usa isso. Que é um rito.* (entrevistada)

Perguntada sobre as cores da capa e sua simbologia, observada a imagem fotográfica (Figura 1), a entrevistada revelou ser uma capa usada desde a época de Florence Nightingale e que perdurou até meados dos anos 1960, sendo que as cores azul por fora e vermelho por dentro eram usadas por professoras em momentos solenes como de Formaturas ou no caso da posse como membro acadêmico na ABRADHENF.

**Figura 1:** Cerimônia de posse na Academia Brasileira de História da Enfermagem

A Figura 1 também revela que a entrevistada portava no busto uma medalha presa por uma fita de cor azul e, perguntada sobre a simbologia desse objeto, ela esclareceu que se tratava de uma medalha na qual estava impressa a imagem da lâmpada usada por Florence Nightingale.

No decorrer da entrevista, ainda foram resgatados outros elementos simbólicos que representavam a enfermagem profissional, por exemplo, o uso da touca:

*Na minha época de estudante, nós tínhamos a cerimônia da touca* [...]*. No segundo semestre do primeiro ano, começava o estágio no hospital. Então, antes de entrar no estágio, as alunas recebiam a touca, então tinha toda uma simbologia. Uma cerimônia importante porque as alunas recebiam a touca das mãos da diretora que punha na cabeça.* (entrevistada)

Continuou o relato do fato e, neste momento, a entrevistada fez uso de nova analogia igualmente significativa em relação à touca: *A diretora da Escola dizia: “vocês vão receber a touca da mesma forma que uma rainha recebe a coroa. Quando ela é coroada rainha e recebe a coroa na cabeça, então vocês vão receber a touca que tem esse mesmo significado”* (entrevistada).

Em outro momento da entrevista, foi resgatada a importância do rito de passagem da lâmpada na cerimônia de formatura dos alunos, sendo que esse mesmo símbolo também se mostrou presente no rito de posse da acadêmica:

*E, no final do curso, tinha a cerimônia, quer dizer, a gente passava, eu recebia a lâmpada no primeiro ano, no final do primeiro ano, da aluna que tivesse tirado a melhor nota passava para a aluna que tivesse tirado a melhor nota no primeiro ano, na média. Então eu recebi a lâmpada das mãos da colega que estava se formando naquele ano, e depois, no final do último ano eu passei a lâmpada para uma aluna do primeiro ano.* [...] *Era uma cerimônia assim, como é que eu diria, é uma coisa histórica na verdade, quer dizer, mas tinha esse significado para nós. Quer dizer, então a enfermeira que se formava, que ela já era enfermeira, passava para a aluna do primeiro ano que estava começando a carreira.* (entrevistada)

Concluiu:

[...] *não é só a capa, quer dizer, então é toda a simbologia porque a gente quer é dar força que a enfermagem é mais do que simplesmente trabalho num hospital, tem essas coisas que eu acho que representam, eu não diria poder, mas representam o nosso esforço como guerreiros da enfermagem. Levar a luz da enfermagem mais adiante, mais para o alto, através destes símbolos.* (entrevistada)

## DISCUSSÃO

A construção da identidade profissional não é um processo isolado, mas sim fruto de uma socialização constante^(1)^. Ela permite que o indivíduo interaja com os grupos sociais, assimilando os valores e a cultura desses grupos e propiciando o surgimento da noção de pertencimento no indivíduo. Afinal, reconhecer-se membro de um grupo social é essencial no processo de construção das identidades profissionais, e isso fica bem claro na fala da entrevistada: *reconhecimento dos próprios colegas* (entrevistada).

O reconhecimento pelo grupo é um elemento imprescindível nas interações sociais e, segundo Dubar^(1)^, o indivíduo, em contrapartida, necessita reconhecer-se dentro desse grupo, para que seja despertado o interesse em fazer parte dele, dando início ao processo de tentar pertencer, movimento que influenciará no processo de construção de sua identidade profissional.

É nas relações sociais que os indivíduos têm sua identidade profissional influenciada e, em contrapartida, influenciam as construções identitárias profissionais dos demais indivíduos: [...] *ela me passou esse exemplo, e eu quero deixar esse exemplo para as futuras gerações. Os novos enfermeiros, os futuros colegas* [...] (entrevistada). Segundo Dubar^(1)^, as construções identitárias são processos dinâmicos e inacabados que operam mais ou menos sob um clima de instabilidade. Essa instabilidade é fundamental para que essas construções sejam questionadas, revistas e reconstruídas a todo momento pelos indivíduos.

No caso da entrevistada, nota-se que o resgate do nome da Profª Amália Correa de Carvalho, personalidade escolhida para nomear a primeira cadeira ocupada pela profª Drª Taka Oguisso, foi uma enfermeira que influenciou a trajetória profissional da acadêmica, e sua escolha resgata e mantém viva a memória e os feitos dessa personalidade como inspiradora para as boas práticas em enfermagem. Hoje, a biografia da profª Taka Oguisso serve de inspiração para seus pares, rendendo-lhe reconhecimento e posição de desta que no grupo social, naquele espaço da Academia, rendendo-lhe pioneirismo em ser o primeiro membro Acadêmico da ABRADHENF.

Sua aprovação por unanimidade, conforme registro em Ata da Assembleia^(12)^, revela que o grupo social endossa e reconhece a relevância desse ator social para a coletividade, e o excerto seguinte demonstra que a homenageada tem conhecimento de sua relevância para o grupo: *Então eu acho que esse é o ponto mais importante* [...] *você aceita e assume uma posição como essa que é uma posição de deferência dos próprios colegas* [...] (entrevistada).

Dessa forma, as construções identitárias profissionais são, necessariamente, intermediadas pela socialização, e os movimentos empreendidos pelos indivíduos na busca por reconhecimento e pertencimento aos grupos sociais são determinantes para os processos de construção de suas identidades.

O rito de posse, pioneiro na ABRADHENF, tem um significado importante para o grupo social ali envolvido, na medida em que foi o primeiro: [...] *nós estávamos seguindo um rito ali, nós estávamos criando um rito para essa cerimônia de designação de um membro acadêmico da Academia* (entrevistada). Essa construção coletiva de um rito de posse demonstra a harmonia envolvendo seus integrantes e reforça a identidade profissional de cada um deles, configurando engajamento no que Dubar^(1)^ chama de “reconhecimento da utilidade social”. Todos ali presentes comungavam a relevância social do rito que estava sendo criado e serviria de modelo para os demais empossamentos.

Pensado, esmerado na preparação, na fina escolha dos elementos integrantes, o rito esteve permeado por símbolos cujos significados atribuídos a cada um deles renovaram o ideário de “ser enfermeiro”, da homenageada e dos demais atores sociais. “Ser enfermeiro” está profundamente relacionado com as construções identitárias profissionais, e Dubar^(1)^ diz que as instituições devem proteger seus integrantes dos riscos de perda de identidade e, quanto maior for o risco, mais elaborados devem ser os ritos.

Nesse contexto, entende-se por rito um conjunto formalizado em certa dimensão simbólica, caracterizado por configuração espaço-temporal, pelo recurso de uma série de objetos, sistemas de linguagens, comportamentos e signos que necessitam ser decodificados, para que possam ser entendidos como bem comum do grupo^(26)^.

O resgate da identidade profissional da enfermagem teve início antes mesmo do próprio rito em si, com a confecção de uma capa que a homenageada deveria usar na cerimônia de empossamento. Não se tratava de uma roupa qualquer, mas sim de um traje usado somente pelas enfermeiras até meados dos anos de 1960. Nesse sentido, ele resgata um símbolo da enfermagem para as novas gerações de enfermeiros alí presentes no cerimonial, pois retrata um momento na história da enfermagem em que essa capa servia como identificador, para os demais profissionais e pacientes, de que aquela profissional era uma enfermeira. A vestimenta conferia à usuária uma identidade profissional inconfundível e, ao mesmo tempo, diferenciadora^(27)^.

A analogia feita pela acadêmica entre a capa usada por ela na cerimônia e as togas usadas pelos juízes revela a simbologia atribuída à roupa e a relevância da pessoa que a veste. Assim como a toga usada pelo juiz o qualifica como autoridade máxima em matéria de Direito, a vestimenta usada pela homenageada a qualifica como autoridade na área da História da Enfermagem. Nota-se aqui a ocorrência de novas representações atribuídas à vestimenta, antes elemento identificador de uma profissional de enfermagem, agora também representação de autoridade constituída, o que corrobora o pensamento de Dubar^(1)^ de que representações não são transmitidas e constituídas de uma vez por todas, elas são construídas sob forma de rearranjos periódicos resultantes de novas assimilações de elementos tomados de diversos setores do entorno.

Outros símbolos da enfermagem foram resgatados pela acadêmica, como a touca e o rito envolvendo sua entrega para a aluna de graduação, bem como o rito de passagem da lâmpada. Todos nacionalmente conhecidos pela enfermagem e que reportam à representação de uma identidade profissional sem equívocos, a enfermagem. Assim, o resgate desses símbolos materiais e imateriais pela acadêmica, neste último caso, a referência à profª Amália e à Irmã Tereza Notarnicola, corroboram para reforçar a identidade da enfermagem e transformar as identitárias profissionais de todos ali presentes, pois, segundo Dubar^(1)^, a socialização se torna um processo de construção, desconstrução e reconstrução de identidades, principalmente na esfera profissional, em que cada um deve aprender a tornar-se ator.

A atmosfera envolvendo a cerimônia de posse da acadêmica produziu efeitos transformadores na identidade profissional da homenageada: “[...] *eu assumo essa gratificação muito mais como uma responsabilidade”* (entrevistada), demonstrando a teoria dubariana de que a identidade profissional é um processo inacabado no qual a socialização se constitui como meio para que tais identidades sejam sempre revisitadas, desconstruídas e reconstruídas. Todos os elementos usados na cerimônia possuem um simbolismo transformador para as identidades ali presentes. A utilização desses elementos no cerimonial corrobora a afirmação de que eles necessitam fazer parte do universo de significado do grupo social para que possam ser decodificados e assimilados como bem comum^(26)^.

A primeira acadêmica foi indicada em 28 de setembro de 2012, conforme Ata da Assembleia Geral^(12)^ da entidade, com base nas normas estabelecidas pelo Estatuto Institucional^(28)^, que estipula a possibilidade de quatro categorias como membros, dentre elas, a de Acadêmico e, pelo Regimento Interno, para Membro Acadêmico^(29)^, que estipula os critérios para a candidatura de um associado a Membro Acadêmico. Nota-se, nesses documentos, a estipulação de hierarquias entre essas categorias de membros, dentre as quais, a de Membro Acadêmico é a que confere maior *status,* portanto, a mais importante a ser alcançada dentro do grupo social. Pertencer a esse grupo seleto desperta, nos demais integrantes, processos de busca por pertencimento que, segundo Dubar^(1)^, são movimentos em que o(s) indivíduo(s) de um determinado grupo busca(m) os meios necessários para pertencer a outro grupo, o de referência, cuja identificação principal no grupo de pertencimento é a que vincula o indivíduo a seu *status.*

Aprofundando mais nesses documentos, o Estatuto Institucional^(28)^ prevê que o membro efetivo poderá ser membro acadêmico caso tenha destaque na produção de pesquisas e publicações na área de História da Enfermagem, segundo critérios de quantidade e qualidade da produção. Percebe-se que há uma valorização da produção intelectual dentro da Academia e, segundo Dubar^(1)^, isso faz com que a pessoa que queira ascender do grupo de pertencimento para o de referência, dentro da instituição, busque a qualificação como meio para ser aceito naquele grupo cuja identificação principal nele é a que vincula o indivíduo aos saberes adquiridos.

Outra questão refere-se aos documentos institucionais e tem relação direta com as construções identitárias profissionais da enfermagem, ou seja, o fato de o Estatuto da Academia^(28)^ permitir a filiação de profissionais ou investigadores não enfermeiros, como historiadores, por exemplo, desde que desenvolvam estudos históricos (Artigo 5º). Corrobora esse posicionamento o Regimento Interno para Membro Acadêmico^(29)^, o qual não estabelece a prerrogativa de que somente enfermeiros podem se candidatar e serem indicados à categoria de membro acadêmico. Com isso, há possibilidade de que membros não enfermeiros possam se candidatar à categoria de membros efetivos acadêmicos, considerando-se a contribuição relevante do candidato e a produção acadêmica na área da História da Enfermagem.

O ingresso de outras profissões na Academia tem implicações positivas para a construção identitária do coletivo da Academia Brasileira de História da Enfermagem, considerando que a construção da identidade profissional, segundo Dubar^(1)^, ocorre pelo processo do olhar para si mesmo e do olhar dos outros por meio das relações sociais.

Para Dubar^(1)^, a reprodução das identidades baseia-se na extrema importância do reconhecimento dentro das relações sociais. O ingresso de um profissional não enfermeiro na Academia poderia levar ao que Dubar^(1)^ chama de “crise do espaço social de reconhecimento”, caracterizado como um ambiente institucional onde as relações sociais podem produzir comportamentos capazes de bloquear a construção de espaços sociais de reconhecimento.

Outro aspecto relevante refere-se aos ritos institucionais, que têm por finalidade criar um ambiente em que as identidades profissionais possam ser reforçadas e protegidas, evitando a perda de identidade^(1)^, mas, para que isso aconteça, todos os elementos, símbolos e signos utilizados no rito necessitam ser decodificados para que possam ser entendidos como bem comum do grupo^(26)^. A entidade precisa garantir que esse profissional não enfermeiro realize esse processo de decodificação de forma adequada, produzindo em si um universo de significados que contribua para a sua construção identitária profissional.

E, por fim, questões envolvendo processos de identificação estão em jogo, tendo em vista que esse elemento é importantíssimo nas relações sociais dentro do grupo ou entre os grupos dentro da instituição. A identificação é que fomenta os desejos dos atores sociais de fazer parte deste ou daquele grupo social, num processo chamado por Dubar^(1)^ de “mobilidade”. Esse profissional não enfermeiro precisa se identificar com o grupo de referência dentro da Academia, o grupo dos membros acadêmicos, para que possa imbuir-se do desejo de pertencer a ele por meio da produção de pesquisas e publicações na área de História da Enfermagem, segundo critérios de quantidade e qualidade da produção^(28)^.

### Limitações do estudo

Este estudo mostra o processo de construção da identidade profissional focado em uma liderança relevante para a enfermagem brasileira.

Tendo em vista que a construção da identidade profissional é individual, apesar de ser influenciada pela socialização, não é possível estabelecer generalizações para as demais construções identitárias dos atores sociais da Academia.

Seriam necessários novos estudos para identificar como ocorrem tais construções identitárias.

### Contribuições para a área da enfermagem

Os achados contribuem para fomentar as discussões em História da Enfermagem e Identidade Profissional, bem como trazem uma relevante contribuição no sentido de reforçar a importância dos ritos institucionais para as construções identitárias profissionais na enfermagem e para o fortalecimento dessas identidades.

## CONSIDERAÇÕES FINAIS

Os ritos, seus signos, símbolos e representações constituem uma parte relevante nos processos que favorecem as construções identitárias profissionais na enfermagem, na medida em que introjetam, no indivíduo, a ideia de pertencimento diante dos grupos sociais dos quais faz parte. Também implicam em reconhecimento de uma trajetória histórica profissional e social deste ator social pelo coletivo.

O rito de posse foi importante por manter viva a memória e o sentido de “ser enfermeiro” na perspectiva da acadêmica aqui referida, renovando seus compromissos com a enfermagem.

É relevante manter-se atento às condições que possam favorecer a perda de identidade, tendo em vista que a construção da identidade profissional é um processo dinâmico, inacabado, no qual as relações sociais interferem a todo momento.

## Data Availability

Os dados da pesquisa estão disponíveis no corpo do artigo.
